# Few negative effects of psychotherapy in a psychiatric day hospital: a follow-up survey to a multiprofessional treatment with acceptance and commitment therapy

**DOI:** 10.3389/fpsyt.2024.1235067

**Published:** 2024-02-08

**Authors:** Christoph Richter, Ronja Rutschmann, Nina Romanczuk-Seiferth

**Affiliations:** ^1^ Clinic for Psychiatry, Psychotherapy and Psychosomatics, Vivantes Klinikum Kaulsdorf, Berlin, Germany; ^2^ Charité – Universitätsmedizin Berlin, Corporate Member of Freie Universität Berlin and Humboldt-Universität zu Berlin, Department of Psychiatry and Neurosciences, Berlin, Germany; ^3^ MSB Medical School Berlin, Department of Psychology, Berlin, Germany

**Keywords:** acceptance and commitment therapy, side effects, negative effects, therapy outcome, day treatment

## Abstract

While there are many studies on psychotherapy and its efficacy – in terms of desired outcomes – there is comparatively little evidence on the possible negative effects of psychotherapy. The aim of this study was to investigate the possible negative effects of a multi-professional psychiatric day hospital treatment for patients with mental health disorders based on Acceptance and Commitment Therapy (ACT), including possible confounding factors. Fifty-one patients with a range of psychiatric diagnoses were assessed three months after an ACT-based psychiatric day hospital treatment. Questionnaires were used to measure negative effects of psychotherapy (INEP), subjective quality of life (WHOQOL-BREF), and symptomatology (BDI-II and SCL-90-R). Correlational analyses and group comparisons were performed to determine the relationship between the sum of reported negative effects on the one hand and symptomology, quality of life, and sociodemographic variables (gender, age, diagnosis, education) on the other hand. At least one negative effect out of a list of 18 possible effects was reported by 45% of participants, and 10% reported more than two. The number of negative effects reported correlates positively with symptomology and negatively with quality of life. The sum of reported negative effects does not correlate with age or gender and does not vary by education level and primary diagnosis. In the light of previous findings, patients included in this study showed lower rates of negative effects, both overall and at item level. Practical implications of these findings are discussed.

## 1 Introduction

While many studies demonstrate the efficacy of psychotherapy in terms of desired effects, evidence of possible adverse or negative effects is comparably rare. This is true for different settings, including hospital treatment, and for different approaches of psychotherapy. For a long time, possible harmful or negative effects of psychotherapy were not systematically recorded at all (1). This shortcoming is made even more apparent when compared to drug trials, where documentation of negative effects is standard practice. Moreover, the demonstration of a good risk-benefit ratio is a prerequisite for the approval of a newly developed drug. In contrast, it remains unclear to what extent psychotherapeutic methods that have been shown to be beneficial for patients may also cause unintended effects or harm (2–4).

There are several possible reasons for the lack of systematic assessment of negative effects in psychotherapy studies, including a lack of awareness in the research community, difficulties in defining negative effects of psychotherapy, a possible publication bias, or methodological problems. Another possible reason is that negative effects in psychotherapy are often attributed to the therapist, so assessing such aspects of psychotherapy may have a threatening quality for many therapists (5).

In order to systematically record unintended effects of a specific treatment, it is important to first define what is meant by them. First, it is important to distinguish whether an adverse event is a coincidental occurrence or is due to the therapy. Second, negative effects should be distinguished from other adverse outcomes of psychotherapy, such as non-response, relapse, deterioration, or discontinuation of therapy. And third, it is important to distinguish between negative effects of correctly applied therapy and outcomes of therapeutic malpractice (1, 6). Previous attempts to record negative effects often did not make these distinctions, leading to the incorrect impression that side effects were the result of therapeutic misconduct and did not occur in correctly applied therapies (1). As discussed above, this may have contributed to the lack of systematic reporting of negative effects for a long time. It is therefore important to emphasize that negative effects do also occur in correctly applied psychotherapy (6). A negative effect of psychotherapy is therefore defined as 1) an adverse event that 2) is causally attributed to the therapy and 3) is not the result of therapeutic misconduct (1).

Available measures of negative effects of psychotherapy mainly include surveys and questionnaires that capture patients’ subjective experiences. The Inventory for the Assessment of Negative Effects of Psychotherapy (INEP; 7) is a questionnaire that assesses negative effects of psychotherapy, as defined above, as well as therapeutic malpractice from the patient’s perspective. It is practical and has been used in several studies with patients with mental health disorders (7–11). In these studies, between 58.7% and 97.6% of the patients reported at least one negative effect, such as having difficulty making important decisions without their therapist, having more downs around the end of therapy, or feeling hurt by the therapist’s statements.

Multi-professional hospital treatment for patients with psychiatric disorders requires specific psychotherapeutic approaches. One evidence-based psychotherapeutic approach that is particularly useful for use in clinical settings is Acceptance and Commitment Therapy (ACT). ACT is a transdiagnostic, process-based psychotherapy that integrates behavioral therapy techniques with mindfulness, acceptance, and value-orientation. It promotes new, healthier behaviors by improving psychological flexibility (12). ACT has become increasingly popular in recent years and has been implemented in a wide range of therapeutic settings, including primary mental health care, and ACT shows growing evidence in terms of randomized controlled trials and meta-analyses, particulary for chronic pain, depression, mixed anxiety, obsessive-compulsive disorder, and psychosis, with modest or strong research support (13).

While many studies demonstrate the efficacy of ACT in terms of desired effects, little is known so far about undesirable or negative effects. The only two studies assessing negative effects of ACT-based treatment were not conducted in patients with psychiatric diagnoses, but in patients with functional somatic syndromes (14) and diabetes (15). Both found a significantly lower frequency of patients reporting negative effects (31% and 38%, respectively) than studies focusing on other therapeutic methods (see above). However, it is questionable whether these results can be generalized to populations with psychiatric diagnoses.

Therefore, the aim of this study is to investigate the reported negative effects of an ACT-based multiprofessional treatment in a psychiatric day hospital. In addition, possible moderating factors such as age, gender, educational level, subjective quality of life (QL), and symptomology will be investigated. Finally, the results will be discussed in comparison to studies in other patient groups, settings, or therapeutic approaches.

## 2 Materials and methods

This study was conducted within the framework of a scientific evaluation trial of an ACT-based multidisciplinary treatment in a psychiatric day hospital (16). The investigation was carried out in accordance with the Declaration of Helsinki (2013). The study was approved by the Ethics Committee of the Medical Association Berlin (12th February 2020, case number Eth-03/20) and was retrospectively registered in the German Clinical Trials Register (http://www.drks.de/DRKS00029992, identifier: DRKS00029992) on August 19th, 2022. All participants in the study gave written informed consent after the nature of the procedures had been fully explained.

Participants were recruited at a psychiatric day hospital in Berlin, Germany, where ACT was implemented as the main psychotherapy approach. Therapies included group psychotherapy (see 17), individual therapy sessions, and an ACT matrix group (see 18), as well as occupational and art therapy, music therapy, movement therapy, and mindfulness training. The entire professional team was trained in ACT and participated in regular ACT-based supervision. Additionally, medication was offered in accordance with medical indication and German Guidelines.

### 2.1 Participants

Of the 92 participants in the evaluation study (see 16), 10 did not agree to follow-up, leaving 82 patients contacted for this survey. Of these, 28 did not respond, and 3 did not complete the Inventory for the Assessment of Negative Effects of Psychotherapy (INEP), leaving a final sample of 51 participants in total. Their mean age was 44.31 years (SD = 12.87, range 19-65) and the mean duration of treatment was 48.88 days (SD = 7.18, range 38-65). The sample characteristics are described in **Table 1**.

**Table 1:** Sample characteristics.

		n	%
Gender			
Male		18	35.3%
Female		33	64.7%
Highest level of education			
Secondary School (“Hauptschulabschluss”)		7	13.7%
Intermediate (“Realschulabschluss”)		29	56.9%
A-levels (“Abitur”)		9	17.6%
Academic degree		6	11.8%
Primary diagnosis			
F2	Schizophrenia, schizotypal and delusional disorders	5	9.8%
F3	Mood (affective) disorders	30	58.8%
F4	Neurotic, stress-related and somatoform disorders	13	25.5%
F6	Disorders of adult personality and behavior	3	5.9%

Diagnoses classified according to the ICD-10 (19).

### 2.2 Measures

Three months after the end of treatment at the psychiatric day hospital, the participants received the following questionnaires: Inventory for the Assessment of Negative Effects of Psychotherapy (INEP), Beck Depression Inventory-II (BDI-II), Symptom Checklist-90-Standard (SCL-90-S), and World Health Organization Quality of Life - Short Version (WHOQOL-BREF). The questionnaires were pseudonymized and patients were assured that none of their information from the follow-up survey would be disclosed to the psychiatric day hospital treatment team.

The INEP consists of 21 questions that are answered by the patient after the end of the therapy. It assesses negative effects in the domains of intrapersonal change, relationships, stigma, emotions, and work, as well as the therapeutic relationship and malpractice (7). The first six items can be answered from +3 to -3, allowing patients to describe both positive and negative changes (e.g., feeling better/worse or having less/more difficulty dealing with the past). In the second part of the INEP, only negative effects can be reported on a scale of 0 to 3 (not at all, a little, partially, completely). For each item in the first and second parts, patients can indicate whether the changes were caused by the treatment or other circumstances, or by both. The sum score and the frequency distribution allow an assessment of the burden of negative effects and a comparison with other studies. The scale is also used to calculate a mean value that indicates the intensity of the identified negative effects (7). The assignment of individual items to scales varies between studies (7, 9, 20, 21). The third part of the INEP does not assess negative effects in the strict sense, but rather the therapeutic relationship and malpractice. The original INEP also includes three questions that capture therapeutic misconduct in the form of potential violations of the law by the therapist. For reasons of data protection and corporate law, these questions were removed from the questionnaire in this study, leaving 18 remaining items.

Current symptomology was assessed using the Symptom Checklist-90-Standard (SCL-90-S) (22). This instrument measures a person’s physical and psychological symptoms on nine different scales. In addition, the Global Severity Index (GSI) reflects the general psychological distress. Only the GSI was used in the analyses.

The Beck Depression Inventory-II (BDI-II) (23) was used to assess the participants’ depressive symptoms. The BDI-II was originally developed to measure the severity of clinical depression but can also be used to assess subclinical depressive symptoms (24).

Subjective quality of life (QL) was assessed using the WHOQOL-BREF (25). The questionnaire measures the perceived QL in the domains of physical and psychological well-being, social relationships, and environment, as well as the perceived global QL.

In addition, participants indicated their gender, education, and date of birth on the questionnaires. Due to standardized questionnaires, only two genders (male/female) could be selected. The primary diagnosis was reported by the respective therapist. In addition, it was noted whether the medication was changed during the treatment (unchanged, applied/increased, switched, stopped/decreased).

### 2.3 Data analyses

First, a descriptive evaluation of the INEP was conducted according to the authors’ instructions (26). Each item in which the participant reported a negative change that they attributed to the therapy (or both therapy and other circumstances) was counted as a negative effect of psychotherapy. The individual sum of negative effects across the items 1-15 was then calculated for each participant. The frequency (sum and percentage) of participants reporting a negative effect for each item was calculated. The mean value was calculated to determine the intensity of the identified negative effect.

We tested possible confounders of the sum of negative effects using SPSS (IBM SPSS Statistics Version 28.0.1.0, RRID : SCR_019096), using Pearson correlation for metric variables (age, BDI-II, GSI, QL), point-biserial correlation for dichotomous variables (gender), and Kruskal-Wallis test for categorical data (primary diagnosis, education, medication change).

## 3 Results

The majority of the patients (65%) reported feeling better than before the therapy, and most of them (88%) attributed their improvement to the therapy (or both therapy and other reasons). In contrast, 19% of the participants reported a worsening, with the majority of them (60%) attributing this solely to other circumstances.

The frequency and intensity of reported negative effects are shown in **Table 2a**, and the distribution of the sum of negative effects is shown in **Table 2b**. A total of 45.1% of the patients reported at least one negative effect, and 9.8% reported more than two negative effects. The most commonly described negative effects were longer periods of feeling bad (23,5%) and feeling dependent on the therapist (15.7%). Similarly, 15.7% reported feeling coerced by the therapist.

**Table 2a:** Frequency and intensity of reported negative effects.

	n	%	M
Any negative effect (INEP 1-15)	23	45.1%	
Feeling worse	4	7.8%	1.75
More difficult to trust others	1	2.0%	2.00
More troubled by past	5	9.8%	2.00
Worse relationship with partner	1	2.0%	1.00
Worse relationship with family	1	2.0%	3.00
Worse relationship with friends	1	2.0%	3.00
Anxious others find out about therapy	2	3.9%	1.50
Problems with insurance	1	2.0%	3.00
More financial worries	4	7.8%	2.00
Feeling dependent on therapist	8	15.7%	1.75
More difficult to make decisions on my own	7	13.7%	2.00
Partner jealous of therapist	1	2.0%	3.00
Longer periods of feeling bad	12	23.5%	2.17
Became worse person	2	3.9%	2.00
Suicidal thoughts for the first time	1	2.0%	1.00
Felt hurt by therapist’s statements	4	7.8%	1.25
Felt personally ridiculed by therapist	2	3.9%	1.50
Felt forced by therapist to do things	8	15.7%	1.25

Absolute (n) und relative (%) frequency as well as intensity (M; scale 0 to +3) of reported negative effects.

**Table 2b:** Distribution of sum of negative effects.

Sum of negative effects	n	%
0	28	54.9%
1	10	19.6%
2	8	15.7%
3	2	3.9%
4	1	2.0%
5	1	2.0%
10	1	2.0%

The distribution of depressive symptoms, symptom severity, and quality of life are reported in the original study (16) and are therefore omitted here.

There was a significant correlation between the sum of negative effects and pre-treatment depressive symptoms, as well as a negative correlation with pre-treatment psychological QL. There was also a significant correlation between the sum of negative effects and depressive symptoms as well as general psychological distress at follow-up. In addition, environmental QL and physical and psychological well-being were negatively correlated with the sum of negative effects. The correlation was significant for all domains except for the quality of social relationships and global QL (see **Table 3**).

**Table 3:** Pearson correlation of sum of negative effects with other measures.

Time	Variable	n	r	*p* (2-sided)
Pre-treatment	BDI-II	50	.32	.02 *
	GSI (SCL-90)	51	.22	.13
	WHOQOL physical well being	49	-.19	.18
	WHOQOL psychological well being	51	-.28	.05 *
	WHOQOL social relationships	50	-.04	.76
	WHOQOL environment	51	-.17	.24
	WHOQOL global quality of life	50	-.13	.38
Follow-Up	BDI-II	51	.46	<.01 **
	GSI (SCL-90)	50	.36	.01 **
	WHOQOL physical well being	51	-.33	.02 *
	WHOQOL psychological well being	51	-.40	<.01 **
	WHOQOL social relationships	51	-.23	.11
	WHOQOL environment	51	-.30	.03 *
	WHOQOL global quality of life	51	-.27	.06

r correlation size, *p ≤.05, **p ≤.01.

Age and gender did not correlate with the sum of negative effects (all *p* >.55).

Overall, 80% of patients received psychopharmacologic medications at some point during treatment. Medication was not changed during treatment in 47% of the cases, was applied/increased in 31%, was stopped/decreased in 14%, and was switched in 8%. These groups did not differ significantly in the number of negative effects reported (*p* = .28).

Similarly, the Kruskal-Wallis test showed no significant difference in the number of reported negative effects between different levels of education (*p* = .08), nor between different primary diagnoses (*p* = .23).

## 4 Discussion

### 4.1 Key findings and interpretation

While positive outcomes of psychotherapy have been the focus of psychotherapy research, potential negative outcomes have been less intensely studied so far. Therefore, this study examined the negative effects of psychotherapy in an ACT-oriented treatment in a psychiatric day hospital.

Most patients in the ACT-based psychiatric day hospital treatment reported to have benefited from the treatment at the three-month follow-up. At the same time, almost half of them reported at least one negative effect of the treatment, with the most commonly reported negative effect being periods of feeling bad (24%). At first glance, this may seem like a high proportion, but these results need to be compared with other studies of negative effects of psychotherapy reported by patients with psychiatric diagnoses. In the original study on the construction of the INEP, Ladwig et al. (7) found that 93.8% of participants reported at least one negative effect. The most commonly reported effect was feeling hurt by the therapist’s statements (55.9%). Abeling et al. (9) found 70.5% reporting at least one negative effect, with the most frequently reported negative effect being longer periods of feeling bad (39.9%). Rheker et al. (8) compared negative effects reported by patients from a psychiatric hospital and a somatic rehabilitation hospital. At least one negative effect was reported by 58.7% of the patients with psychiatric diagnoses and by 45.2% of the patients with somatic diagnoses. The most common negative effect was periods of feeling bad (31.2% and 30.2%, respectively). In an inpatient CBASP treatment for chronic depression, 92.3% of patients reported at least one negative effect, with the most common negative effect being feelings of dependence on the therapist (45.2%) (10). In a DBT-oriented psychiatric day hospital for patients with borderline syndrome, 97.6% of the patients reported at least one negative effect with the most prevalent effect of longer periods of feeling bad (97.6%) (11). Thus, it can be concluded that patients in the current study experienced negative effects they associated with the ACT-based psychiatric day hospital treatment, but at a significantly lower level – both in total and at the item level – than might have been expected in light of previous findings. This is all the more surprising given that part of the therapeutic strategy in ACT to normalize and even evoke negative thoughts and feelings in order to learn alternative ways of dealing with such adverse experiences.

Regarding previous findings on negative effects of specific ACT-based treatments, studies in patient populations with functional somatic syndromes and diabetes (14, 15) had shown slightly fewer negative effects than those found in this study. This suggests that the nature of the patients’ symptoms and the clinical picture may influence the occurrence of negative effects. This hypothesis is supported by our finding that higher pretreatment depressive symptoms and lower pretreatment psychological well-being correlated with the sum of negative effects reported by the patients at follow-up. Apart from this, it remains unclear whether psychotherapy for patients with psychiatric disorders in a psychiatric day hospital setting can be compared at all with psychological interventions for patients with somatic diagnoses.

Furthermore, we found that the item “I felt forced by the therapist to do something” was reported with a comparably high fequency (15.7%). In most other studies, the rate for this item ranged from 3.2% (8) to 10% (9), but a rate of 31.3% has also been previously described (7). One explanation for the relatively high occurrence rate of this item in our sample is that this effect may be driven, at least in parts, by ACT-specific features. A specific goal of ACT-oriented treatments is to help patients develop a concrete commitment, so that they can have new experiences and live a richer, more value-oriented life. This repeated focus on commitment is likely to be experienced as coercion. Another relevant aspect is the influence of contextual factors here. The COVID pandemic had started at the same time as our data collection, so patients were required to wear masks, keep their distance, and undergo regular COVID testing during treatment. This may have increased sensitivity to feeling coerced. Furthermore, it should be considered that some authors count the item “felt forced by the therapist to do something” as therapeutic misconduct rather than as a negative effect of psychotherapy (7, 10, 11), while others assign it to the therapeutic relationship (9, 21). In both cases, the item does not contribute to the total sum of negative effects (26).

The number of negative effects reported by the patients was also associated with higher symptom severity and lower quality of life at post-treatment. One explanation for this effect is that negative effects of treatment are associated with a lower response to therapy or even deterioration in general. Abeling et al. (9) showed that the more expectations of psychotherapy were met and the more positive the general therapy success was judged, the lower the number of negative effects. It should also be noted that there is a content overlap between the phenomena measured by the respective items of the SCL-90-S and the BDI-II as well as the item that contributed most to the total sum of negative effects (“longer periods of feeling bad”).

As a general factor, the influence of the therapeutic setting on our results must also be considered. On the one hand, the focus of the treatment in the day hospital was primarily group therapy, which is known to be particularly prone to negative effects (27). On the other hand, day hospital patients reported fewer negative effects than inpatients in a previous study (9). Other factors that were not part of this research may also have influenced the perception of negative outcomes, such as high treatment expectations, patient motivation, therapeutic alliance, coping strategies, etc. (9, 28), and should be systematically examined in future studies in their influence on the reporting of negative effects. Finally, the general discourse on whether negative effects should be avoided or whether they are an unavoidable or even necessary part of psychotherapy is ongoing (29).

It is reasonable to assume that ACT, like other psychotherapy approaches, will have negative effects. Potential negative effects need to be considered as part of the professional routine in psychotherapy. Patients with higher symptom severity and lower quality of life post-treatment may be more likely to experience increased negative effects in a retrospective, subjective survey. Further research is needed to identify the relevant conditional variables.

### 4.2 Limitations and strengths

There are several methodological limitations to the current study: First, the use of a retrospective, subjective survey carries the risk of a general recall bias as well as mood-congruent effects, i.e., patients who feel relatively well three months after therapy also report fewer side effects than others. Another important limitation is the lack of a control group in this naturalistic study setting. Thus, although patients reported substantially fewer negative effects than in comparable studies, we cannot be certain that this is an outcome related to the ACT-based psychotherapeutic treatment. Comparative studies would be necessary to investigate whether ACT-based treatments actually have fewer negative effects than other interventions. Also, population- or setting-specific effects cannot completely be ruled out here. Further, especially in day-care settings there are several factors contributing to the overall treatment effect, like the selection process of suited patients for the day clinic, the community of the patients, relief from everyday life challenges, additional creative therapies, etc. Thus, population- or setting-specific effects cannot completely be ruled out here. Since in the end a total of 51 patients out of 92 patients were available for data acquisition, it is to mention that we also cannot rule out any more selection effects here.

When interpreting the results, it is to consider that some items in the INEP directly refer to the symptoms. Thus, little change in symptoms in patients interferes with the perceived level of negative effects in terms of aggregating measures. This could also be an explanation for part of the correlations we have found.

Strengths of this study are, first, that patients in this study were able to provide information anonymously without fear of compromising the therapeutic relationship. Furthermore, we examined a specific psychotherapeutic program (ACT) in a defined multiprofessional psychiatric day hospital setting and also analyzed correlations with quality of life and intensity of psychological and somatic symptoms at the time of the survey. To our knowledge, this is the first study to examine the negative effects of ACT in a psychiatric setting.

## Data availability statement

The raw data supporting the conclusions of this article will be made available by the authors, without undue reservation.

## Ethics statement

The studies involving humans were approved by Ethics Committee of the Medical Association Berlin. The studies were conducted in accordance with the local legislation and institutional requirements. The participants provided their written informed consent to participate in this study.

## Author contributions

All authors contributed to the conception and design of the study. RR performed material preparation, data collection and analysis. CR wrote the first draft of the manuscript, and all authors commented on previous versions of the manuscript. All authors contributed to the article and approved the submitted version.
